# Eight complete genome sequences of bacteria isolated from laboratory stock of giant kelp gametophytes

**DOI:** 10.1128/MRA.00984-23

**Published:** 2024-03-15

**Authors:** Bernadeth Tolentino, Sergey Nuzhdin

**Affiliations:** 1 University of Southern California, Los Angeles, California, USA; Montana State University, Bozeman, Montana, USA

**Keywords:** microbiome, macrocystis, kelp, giant, epibiont, pyrifera, bacteria, isolate

## Abstract

We report whole-genome sequences of eight bacteria isolated from laboratory-kept *Macrocystis pyrifera* gametophytes. The bacterial culture collection is maintained in cryostorage and will be utilized in future applications as inoculants. The genomes were assembled using Oxford Nanopore Technology long-read sequencing.

## ANNOUNCEMENT

*Macrocystis pyrifera* is a valuable environmental, ecological, and economic resource (1, 2); however, changing environmental factors have led to a decline in kelp survival (3). The kelp microbiome has been shown to play a significant role in its growth and development (4); bacterial presence at the gametophyte stage has a strong correlation with increased adult biomass (5). Understanding this bacterial community and its genomic makeup is instrumental in developing inoculants for the enhancement of the growth and resilience of *M. pyrifera*. We announce the genome sequences of eight bacteria isolated from *M. pyrifera* gametophytes to establish a culture collection for future inoculant testing.

Laboratory kelp gametophyte strains collected from natural kelp beds located off the coast of Leo Carillo and the Channel Islands were chosen for bacterial isolation based on the highest adult biomass (5). Whole gametophytes ~1 mm in diameter were placed in a sterile 1.5 mL Eppendorf tube and washed 3 times using 0.22 μm filtered artificial sea water and Provassoli’s Enriched Seawater (FAWS + PES) to remove loosely associated microbes (6). The remaining liquid was aspirated from the tube, and a Fisherbrand Pellet Pestle cordless motor was used to fragment the gametophyte. Five hundred milliliters of FASW + PES was added to the pulp and triturated to ensure the homogeneity of the mixture. Twenty milliliters of this solution was plated onto sterile agar plate media made using 1 L of FASW + PES, 4 g yeast extract, 4 g glucose, and 15 g agar, which was parafilmed and incubated at 13°C for 7 days. Individual colonies were subsequently isolated by picking and re-streaking on the same medium three times. One hundred forty isolates were selected for 16S rRNA gene sequencing. Universal 16S rRNA gene primers 27F (5′-AGAGTTTGATCCTGGCTCAG) and 1492R (5’- GGTTACCTTGTTACGACTT) were used for colony PCR. BLASTn alignment of the 16S rRNA gene amplicons against NCBI’s 16S ribosomal RNA sequences (Bacteria and Archaea) database was used to identify eight isolates unique relative to one another, which were grown in respective liquid media for 48 h at 25°C. Bacteria were pelletized through centrifugation and genomic DNA was extracted using the Zymo Quick-DNA miniprep Plus kit and sequenced with Oxford Nanopore technology (https://www.plasmidsaurus.com/). Amplification-free long-read sequencing libraries were prepared using the Oxford Nanopore V14 chemistry kit and sequencing was performed with PromethION R10.4.1 flow cells, generating 210 Mb of raw sequencing data. ONR-Gyppy-for-promethion v6.5.7 was used for base-calling. Read quality was assessed using Filtlong v.0.2.1 and Flye v2.9.1 (7) was used for assembly and coverage calculation. The NCBI Prokaryotic Genome Annotation Pipeline (8) was used for annotation, and CheckM v1.2.2 (9) was used to assess quality and completeness of the assembly. Default parameters were used except where otherwise noted.

Genome statistics are reported in Table 1. Sizes ranged from 3.2 to 4.7 Mbp with GC content of 35%–62%. All genomes were circularized, and several had circularized extrachromosomal elements. Completion was verified through Flye v2.9.1 using Bandage v0.8.1 output (10). GTDB-k v2.3.2 was used for taxonomic classification. We identified *Alteromonas sp018100795, Epibacterium scottomollicae, Sulfitobacter dubius, Cobetia amphilecti, Nonlabens ulvanivorans, Marinobacter salaries, Sulfitobacter sp001634775,* and *Thalassospiralucentensis_A* as the closest related genomes using FastANI v1.3 (11).

**TABLE 1:** . Genome Statistics of Eight Isolated Bacteria from Macrocystis pyrifera Gametophytes

Collection ID	3E.13.1	2E.20.3–1	JM3A4	AH3A4	3E.20.3	3E.34.5	2E.13.4	2D.20.1
Closest Reference Genome (ANI)	Alteromonas sp018100795	Epibacterium scottomollicae	Sulfitobacter dubius	Cobetia amphilecti	Nonlabens ulvanivorans	Marinobacter salarius	Sulfitobacter sp001634775	Thalassospira lucentensis_A
Fasti_ani ID %	98.0736	97.6985	97.025	97.2067	97.4102	94.8018	98.2787	97.2817
No. of reads	48,063	35,838	66,028	106,622	43,965	51,994	56,468	30,201
Read Nv50 (bp)	12,034	14,423	11,243	6,941	12,525	9,859	15,062	12,256
Raw coverage (x)	66	55	100	96	75	58	106	31
Assembled cvg (x)	63	43	99	91	71	57	102	30
Genome Size (bp)	4,662,912	4,214,297	3,191,777	4,132,234	3,200,504	4,632,059	3,532,795	4,566,725
Total DNA (bp)	4,662,912	4,703,457	3,835,953	4,140,784	3,200,504	4,632,059	4,271,872	4,733,519
No. of contigs	1	6	5	2	1	1	8	2
No. of additional plasmids	0	5	4	1	0	0	7	1
GC Content %	43.62	60.81	60.18	62.61	35.25	56.93	60.98	54.89
No. of genes	4,055	4,486	3,827	3,526	2,894	4,369	4,245	4,436
Completeness%	99.84	99.53	99.68	99.14	98.54	100	99.68	100
Contamination %	0.51	0	0.48	0.6	0.46	0.07	0.53	0
Gametophyte ID	CI.11.F.A3	LC.270.F.C4	CI.11.F.A3	CI.11.F.A3	CI.11.F.A3	CI.11.F.A3	LC.270.F.C4	LC.270.F.C4
Accession Number	SAMN37594963	SAMN37594964	SAMN37594965	SAMN37594966	SAMN37594967	SAMN37594968	SAMN37594969	SAMN37594970
SRA	SRS19107619	SRS19107620	SRS19107621	SRS19107622	SRS19107623	SRS19107624	SRS19107625	SRS19107626

## Data Availability

The complete genomes of these strains have been deposited at GenBank under the BioProject number PRJNA1022162 with the following accession numbers: SAMN37594963, SAMN37594964, SAMN37594965, SAMN37594966, SAMN37594967, SAMN37594968, SAMN37594969, SAMN37594970. The raw sequencing reads have been deposited in the Sequence Read Archive under the following accession numbers: SRS19107619, SRS19107620, SRS19107621, SRS19107622, SRS19107623, SRS19107624, SRS19107625, SRS19107626.
